# Vacuum-free transparent quantum dot light-emitting diodes with silver nanowire cathode

**DOI:** 10.1038/srep12499

**Published:** 2015-07-22

**Authors:** Pengtao Jing, Wenyu Ji, Qinghui Zeng, Di Li, Songnan Qu, Jia Wang, Dandan Zhang

**Affiliations:** 1 State Key Laboratory of Luminescence and Applications, Changchun Institute of Optics, Fine Mechanics and Physics, Chinese Academy of Sciences, 3888 Dongnanhu Road, Changchun 130033, China; 2 Department of Physics, Jilin University, Changchun 130023, People’s Republic of China; 3 Jiangsu Key Laboratory for Carbon-Based Functional Materials and Devices, Institute of Functional Nano and Soft Materials (FUNSOM), Soochow University, Suzhou 215123, China

## Abstract

Efficient transparent quantum-dot light emitting diodes (QD-LEDs) are demonstrated by using a silver nanowire (AgNW) cathode. The devices are fabricated through a solution technique, not any vacuum processes are involved. Almost identical performance is obtained for both sides of the transparent device, which is primary due to the high transmittance of AgNW cathode. The maximum luminance (efficiency) for ITO and AgNW side is 25,040 cd/m^2^ (5.6 cd/A) and 23,440 cd/m^2^ (5.2 cd/A), respectively. The average specular transmittance of the device (involving the glass substrate) is over 60% in the visible range. This study indicates that AgNW electrodes can serve as a cost-effective, flexible alternative to ITO, and thereby improve the economic viability and mechanical stability of QD-LEDs. All the results suggest that this is an important progress toward producing transparent QD-LEDs based displays and lighting sources.

During the last two decades, semiconductor quantum dots (QDs) are much attractive as light-emitting materials owing to their unique potential properties, including tunable emission from UV to near infrared, pure emission color, low-cost solution processes, and high efficiency^1^,^2^,^3^,^4^,^5^,^6^,^7^,^8^. Therefore, QDs light emitting diodes (QD-LEDs) are being pursued with much anticipation as viable alternatives to GaN based LEDs and organic LEDs, which dominate today’s market. Further, QD-LEDs exhibit significant advantages over liquid crystal displays and organic LEDs^8^,^9^,^10^,^11^,^12^, including wide gamut and saturated emission. Since the first report on the QD-LEDs^3^, great effort has been devoted to achieving highly efficient QD-LEDs through a variety of means and there has been numerous progress in improving the device performance by optimizing both QD materials and device architectures^13^,^14^,^15^. Recently, some record performance parameters for QD-LEDs, such as maximum luminance over 200,000 cd/m^2^ and high external quantum efficiency of approximately 20.5%, have been reported with an organic/inorganic hybrid device structure^13^,^16^. However, there are still some critical roadblocks, as achieving commercial success will likely require the exploitation of key strengths, such as non-vacuum roll-to-roll type manufacturing and opportunities in flexible applications. As common organic LEDs, the vacuum thermal evaporation is often required to obtain organic active layer and metal electrode in QD-LEDs fabrication processes. The high vacuum techniques are expensive and complicated, which increases the cost in mass production and limits the commercialization process of organic LEDs and QD-LEDs. In order to meet the commercialization requirement, the manufacturing cost of the QD-LEDs must be dramatically decreased. At present, the organic/inorganic functional layers used in QD-LEDs, such as poly(N,N’-bis(4-butylphenyl)-N,N’-bis(phenyl)benzidine) (poly-TPD)^7^, Poly[(9,9-dioctylfluorenyl-2,7-diyl)-co-(4,4’-(N-(4-sec-butylphenyl) diphenylamine)] (TFB)^17^,^18^, Poly(9-vinylcarbazole) (PVK)^16^,^19^, ZnO nanopartilces^13^, and sol-gel TiO_2_^20^, can be obtained by a low-cost solution process. However, the cathode is still deposited via thermal evaporation in a high-vacuum chamber. Recently, an electronic material, a random silver nanowire (AgNW) network film, has been explored for photoelectric applications^21^,^22^,^23^,^24^,^25^ where low sheet resistance (Rs) and high optical transparency (T) in the visible and infrared spectral range are required. AgNW film are fairly stable and can be prepared with excellent control regarding wire geometry, including nanowire length and diameter^26^. It has been reported that AgNW film with highly average transmittance of 85% and low Rs of 10 Ω sq^−1^can be achieved using a simple drop casting technique^27^. We can anticipate that AgNW film should be a suitable alternative electrode in vacuum-free QD-LEDs.

In this study, we fabricated transparent QD-LEDs through all-solution techniques and not any vacuum processes were involved. The charge carriers are injected into the QD-LEDs through an ITO anode and an AgNW cathode. The device performances, including luminance, efficiency and electroluminescence (EL) spectra, are nearly identical for both sides of the transparent QD-LEDs. The maximum luminance (efficiency) for ITO and AgNWs side is 25,040 cd/m^2^ (5.6 cd/A) and 23,440 cd/m^2^ (5.2 cd/A), respectively. Our work offers a simple, reliable and cost-effective approach to fabricate transparent QD-LEDs. These results lay the foundation for rational design of QD-LED structure and offer a practicable platform for the realization of transparent QD-based displays and lightings.

## Results

**Figure 1:**
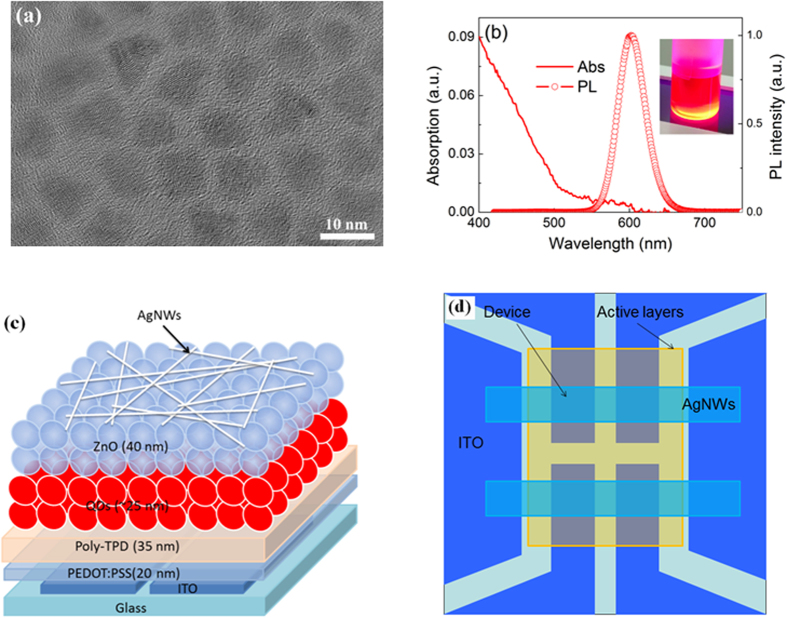
(**a**) TEM image and (**b**) PL and absorption spectra of the QDs. (**c**) The schematic structure and (**d**) top-view of the QD-LED.

Figure 1a shows the representative TEM image of the CdSe/CdS/ZnS QDs. High crystallinity of individual QDs is obtained and the average diameter of the QDs is ~8.0 nm with a 3.0 nm CdSe core, 1.0 nm CdS and ~1.5 nm ZnS shells and these spherical shaped QDs have relatively uniform size distribution. Moreover, no obvious boundary exists between the core and shell as seen in Fig. 1a, which indicates that alloy transition layer should be formed at the CdSe/CdS and CdS/ZnS interfaces of the QDs, leading to a continuous composition gradient inside the QDs. The absorption and PL spectra of the QDs (in toluene) used in our devices are shown in Fig. 1b. The PL peak is located at 602 nm and the full-width at half-maximum (FWHM) is 42 nm. Inset is the photograph of the QD solution exposed to UV light and we can see that vivid red emission is achieved. The quantum yield of CdSe/ZnS core−shell QDs is 60% in solid powder form measured with an integrating sphere. The structure of the devices is schematically shown in Fig. 1c and the QD-LEDs have a typical multilayer structure consisting of QD emission layer sandwiched between a ZnO nanoparticle electron transport layer (ETL) and poly-TPD hole transport layer (HTL) and the AgNW film is used as the cathode deposited by a spin-coating process. Figure 1d is the top-view of a transparent QD-LED. The pixel of the QD-LED consists of the overlapping with an area of 9 mm^2^ between ITO anode and AgNW cathode. Four devices are fabricated on one substrate simultaneously with the same structure.

**Figure 2:**
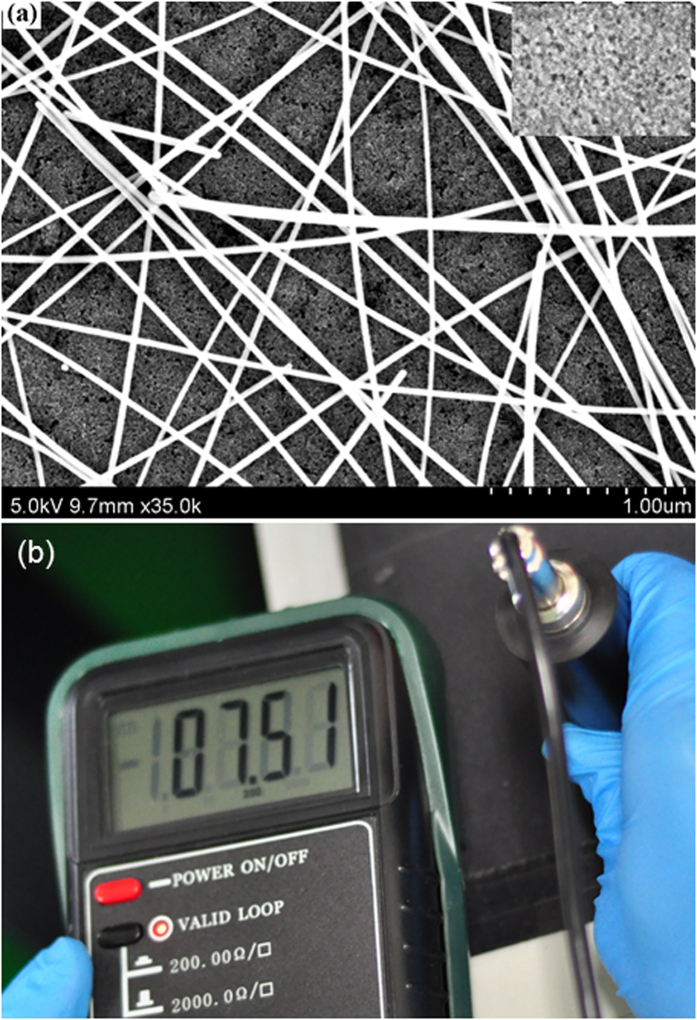
(**a**) SEM image of AgNW cathode. Inset is the SEM image of ZnO before depositing AgNW cathode. (**b**) The photograph of the sheet resistance measurement.

The AgNW film exhibits random meshes over the area of the device as observed from Fig. 2a, indicating no significant bundling between AgNWs, and the length and diameter of NWs are up to thirty micrometers and fifty nanometers, respectively. As is well known, the long metal NWs can form an effective electron percolation network with superior photoelectrical performance than the shorter ones. We measured the Rs of AgNW film by four-point probe. Figure 2b shows the result of measurement and an Rs of 7.51 Ω/square is obtained, which indicates that the AgNW film can be used as the electrode due to their good conductive performance.

**Figure 3:**
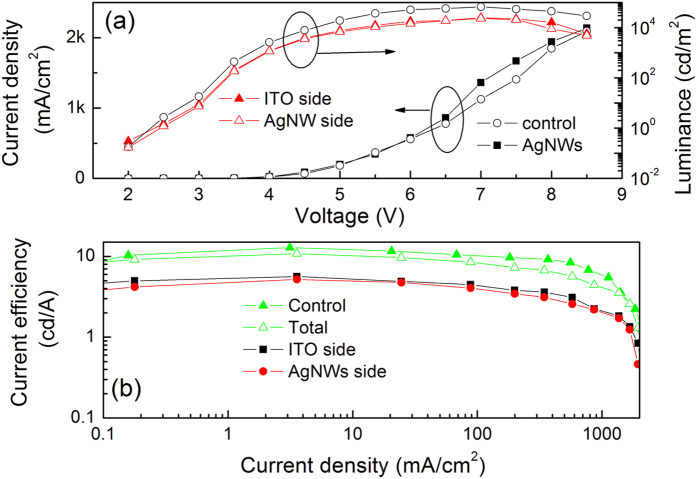
(**a**) Current density-voltage-luminance properties and (**b**) current density-efficiency curves of the transparent QD-LED, as well as the conventional QD-LED with 100 nm Al as the cathode for control.

**Figure 4:**
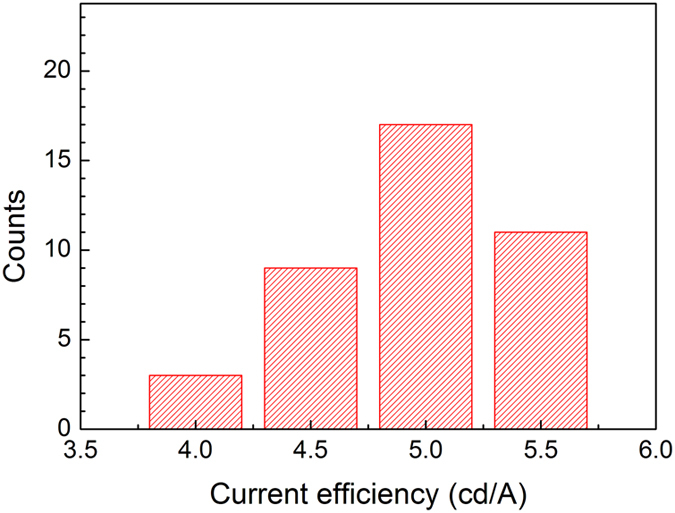
Histogram of current efficiencies measured from 40 devices. The average current efficiency is 5.0 cd/A.

The current density-voltage-luminance properties of the transparent QD-LED are shown in Fig. 3a. It can be seen that the turn-on voltage (here, the turn-on voltage is the applied voltage when the luminance of device is 1 cd/m^2^) for this transparent QD-LED is less than 2.5 V, which indicates the efficient injection of holes and electrons into the QDs to form excitons. Note that there is only a little difference in luminance for both sides of the device. The maximum luminance for ITO and AgNWs sides is 25,040 cd/m^2^ and 23,440 cd/m^2^, respectively, as seen from Fig. 3a. This is contributed to the high transmittance of AgNW film in the devices and their optical properties will be discussed below. A conventional QD-LED having the same device structure to the transparent device but employing Al cathode instead of AgNWs is also built and similar electrical properties to that of AgNWs based device is observed in Fig. 3a, which further indicates a good conductivity of AgNW cathode. Figure 3b shows the current density-efficiency characteristics of the transparent QD-LED, as well as the control device with Al as the cathode. Likewise, almost identical efficiencies are achieved for both sides of the transparent QD-LED. For example, the maximum current efficiencies are 5.6 cd/A and 5.2 cd/A for ITO and AgNW sides, respectively. In addition, the total efficiency of both AgNW and ITO sides of the transparent QD-LED is also similar to that of the control device. The peak total efficiency of the transparent device is 10.8 cd/A relative to 12.9 cd/A for the reference device. To the best of our knowledge, these are the best results for transparent QD-LEDs reported until now^28^. These results further demonstrate that the AgNW film is competent to the electrode in QD-LEDs. In addition, an high reproducibility of AgNWs based transparent QD-LEDs is very important to demonstrate the validity of our results. Figure 4 shows the histograms of current efficiencies (AgNW side) of 40 devices from 4 batches, yielding an average efficiency of 5.0 cd/A. The good reproducibility of the devices demonstrates the feasibility of AgNWs as the cathode in the transparent QD-LEDs.

**Figure 5:**
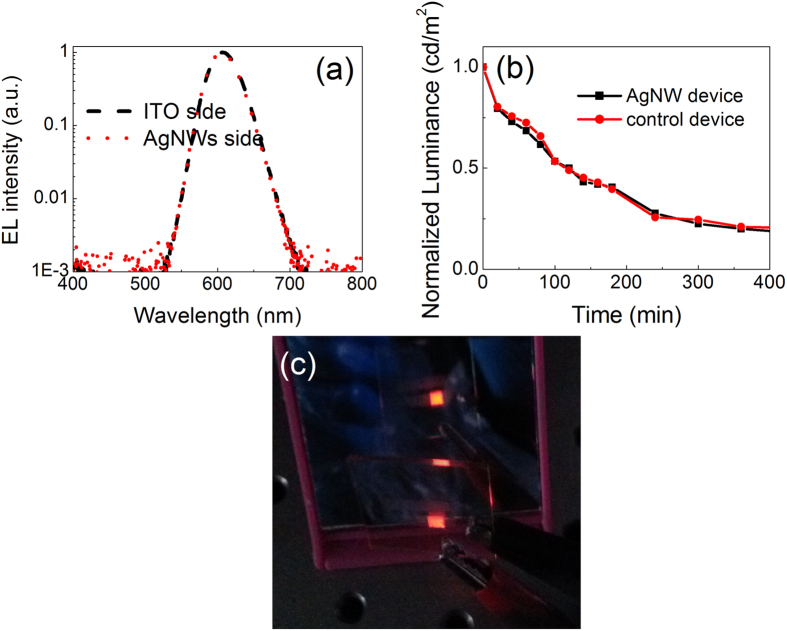
(**a**) EL spectra of the transparent QD-LED for both AgNW and ITO sides. Half-log coordinates is used to clear show the EL components from the QD-LED; (**b**) Photograph of the QD-LED operated at 3.2 V in front of a mirror. The image in the mirror is AgNW side of the device; (**c**) The stability of devices as a function of operating time in air.

Figure 5a shows the EL spectra of the transparent QD-LED for both AgNW and ITO sides at the operating voltage of 5 V. The emission spectrum is the same for both sides of the devices, which further demonstrates the outstanding optical transmittance of the AgNW film. The half-log coordinates are used to clearly exhibit the different components of EL spectra emitted from QD-LED. We can see that a color-saturated and pure red emission completely originating from QDs is achieved in the transparent QD-LED. Note that no emission at the low-energy region in the EL spectra is observed, which demonstrates an efficiently radiative recombination efficiency originating from the band emission of QDs. Moreover, the commonly parasitic EL emission from the adjacent organic layers is also not emerged, which indicates that the excitons are dominantly formed in QDs and effectively radiative recombination occurred. The EL spectrum peak is 606 nm and the FWHM is 43 nm, which is consistent with the PL spectrum with a 602 nm emission peak and 42 nm FWHM as shown in Fig. 1b. In addition, the same EL spectrum peaks for both AgNW and ITO sides are observed, which further demonstrates the high transmittance of AgNW cathode. Figure 5b is a photograph of transparent QD-LED in front of a mirror at the driving voltage of 3.2 V, which intuitively exhibits an identical working device (AgNW side) in the mirror to the top device (ITO side). The devices fabricated in this work are not optimized. There is still room for further improving the QD-LED device performance through the optimization of QD structures, device architecture, fabrication conditions, AgNW electrode properties (such as the length and diameter of AgNWs), and quality of ZnO nanoparticles. Moreover, the device architecture could also be applied to other color QD-LEDs, such as blue and green devices, to fabricate efficient transparent three primary color QD-LEDs for the application in flat panel displays and lighting sources. We also measured the lifetime of transparent and control devices in atmosphere condition without any encapsulation for the devices. The lifetime measurement is implemented under the same applied current for these two devices in order to obtain a more fair result because they have the same device structure and similar I-V characteristics. The measurement is carried out at a constant operation current density of 80 mA/cm^2^, corresponding luminance is 3651 and 9640 cd/m^2^. The results indicate that the AgNW based device has the similar stability to that of the control device as shown in Fig. 5c.

**Figure 6:**
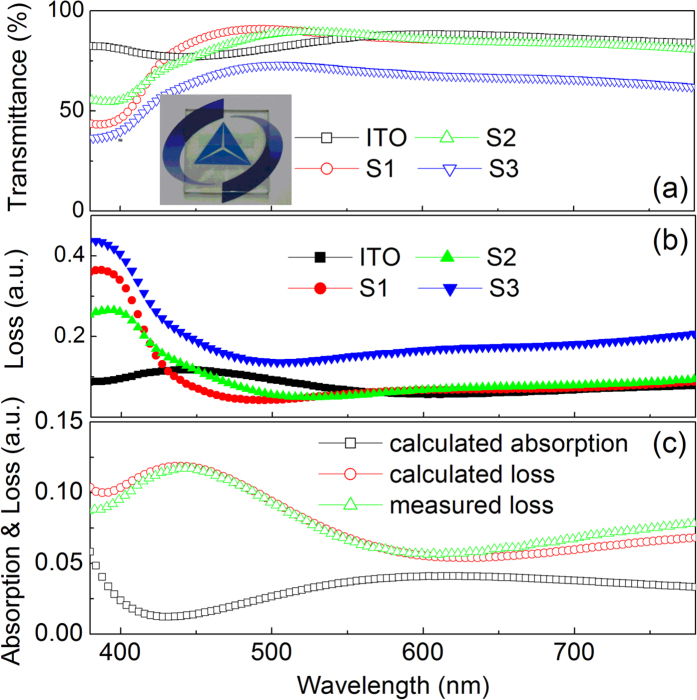
(**a**) Specular transmittance and (**b**) loss of the samples with the structure of glass/ITO/PEDOT:PSS/poly-TPD for S1, glass/ITO/PEDOT:PSS/poly-TPD/QD for S2, and glass/ITO/PEDOT:PSS/poly-TPD/QDs/ZnO/AgNWs for S3. Inset of (**a**) is the photograph of the transparent device. (**c**) The calculated and measured loss for the ITO substrate, as well as the calculated absorption spectrum.

The specular transmittance of glass/ITO substrate and the samples with the structure of glass/ITO/PEDOT:PSS/poly-TPD (named as S1), glass/ITO/PEDOT:PSS/poly-TPD/QD (named as S2), and glass/ITO/PEDOT:PSS/poly-TPD/QDs/ZnO/AgNWs (the transparent device, named as S3) is shown in Fig. 6a. As can be seen, similar specular transmittance is obtained for the glass/ITO, S1, and S2, which is attributed to the low absorption of the organic film and QDs layer. It is worth noting that the specular transmittance of S3 (i.e. the transparent device) is lower than the samples without AgNWs, which should be due to the intense light scattering induced by AgNWs. The inset of Fig. 6a is a photograph of the transparent QD-LED, the device is sufficiently transparent that a Logo of *Changchun Institute of Optics, Fine Mechanics and Physics, (CIOMP) Chinese Academy of Sciences* under the device can be clearly seen. We can see from Fig. 6b that a larger light loss (the loss is the total of absorption and reflection obtained from the transmittance data for the samples) at the short wavelength range is observed for S2 and S3, which is due to the absorption of poly-TPD. The loss near 400 nm is decreased with the QDs deposited on the poly-TPD layer, which may be due to the damage to the poly-TPD induced by the QD depositing process. And the slight increase of the loss for S2 compared to S1 at the range from 430 nm to 500 nm should originate from the absorption of QDs. Another interesting phenomenon is the lower loss of S1 and S2 than that of glass/ITO at the wavelength range from 430 nm to 500 nm. This can be rationalized by the low absorption and high reflectance for the ITO substrate as shown in Fig. 6c. The calculated loss is agreement well with the measured data, which demonstrates the accuracy of the calculation. According to our calculation results, the reflection of the ITO is much higher than the absorption that can be negative. Therefore, when the PEDOT:PSS, poly-TPD, and QD layer were deposited, the reflectance of ITO was decreased due to the antireflection film effect, which was also agreement well with the results shown in Fig. 6a that, compared to glass/ITO, higher transmittance for S1 and S2 was achieved at the region between 430 nm and 500 nm. This further indicates the antireflection film effect is occurred as the deposition of PEDOT:PSS, poly-TPD, and QD layers. When the ZnO and AgNWs layer are deposited on the QDs, the loss is increased over all the visible range, which is attributed to the light scattering rather than absorption of AgNWs and ZnO (the absorption edge of ZnO nanopartilces locates at 360 nm). As reported by other group^29^,^30^, over 10% haze for the AgNW film could be obtained, which would lead to the increase of loss for AgNWs-containing sample in our absorption measurement. We cannot measure the haze of the AgNW film because of the limited equipment in our lab. However, the light scattering effect of AgNW film is a very usual and demonstrated phenomenon^26^,^29^,^30^.

## Discussion

As we know, the length of AgNW is a key factor to affecting the sheet resistance, the longer AgNWs, the lower sheet resistance. The average length of AgNW is around 30 μm used in the cathode, which leads to a high conductive AgNW film. However, it is worth noting that the area of AgNW film also influence the resistance measurement results. A larger AgNW film will lead to more convincing results. In our work, the size of the substrate is 2.5 cm × 2.5 cm. The lower resistance of AgNW film compared with the results reported by other group^29^ may be due to the small substrate size. It is well known that the area of AgNW film dramatically influences the resistance measurement results with a four-probe method. In fact, a larger substrate is also used in our experiments, but the uniformity of the AgNW film deposited by spin-coating processes is very bad. So the 2.5 cm × 2.5 cm substrates were used in our work. In order to characterize the AgNW electrode conductivity, the glass substrates coated with a ZnO nanoparticle layer is adopted because the ZnO layer is also used as the electron transport layer in the QD-LEDs and the sheet resistance is in the range of 7.32 to 8.15 Ω/square. In other words, the composite ZnO/AgNWs layer actually acts as the cathode in the devices. The resistance of AgNW film on glass substrates is not measured due to the different conditions for the AgNWs deposited on glass and glass/ZnO nanoparticle substrates, which will result in a different density of AgNWs on the substrates and can not exhibit accurate results for the sheet resistance measurements.

In conclusion, we have successfully fabricated a transparent red QD-LED with AgNWs as the cathode. The performances of the transparent QD-LED for both sides are almost identical due to the excellent photoelectrical properties of the AgNW film. The sheet resistance of the AgNW cathode is very low, 7.32 to 8.15 Ω/square and the specular transmittance of the transparent device is over 60% in the visible range. The luminance is over 20,000 cd/m^2^ and current efficiency is higher than 5.2 cd/A for both sides of the device. To the best of our knowledge, our present work demonstrates the best-performing all-solution-processed transparent red QD-LEDs. Although only red QD-LEDs were fabricated, it is evident that there are no fundamental limits of extending these to other color (for example, blue, green, and yellow, etc.) QD-LEDs These results open a way for the fabrication of transparent QD-LEDs with optimum and identical performance for both sides and demonstrate a remarkable progress in transparent QD-LEDs, which offers a practicable platform for the realization and commercial production of QD-LED based displays and lightings.

## Methods

### Preparation of CdSe/CdS/ZnS QDs, ZnO nanocarystals and AgNWs

CdSe/CdS/ZnS QDs and ZnO nanoparticles were prepared according to that reported in litherature^31^. Silver nanowires were synthesized according to a reported procedure and KBr was also used to obtain longer AgNWs^29^.

### Device Fabrication

The The QD-LEDs with a structure of ITO/PEDOT:PSS (~20 nm)/poly-TPD (~35 nm)/QDs(~35 nm)/ZnO (~40 nm)/AgNWs were built. Layers of PEDOT:PSS, poly-TPD, QDs, and ZnO are used as hole-injection layer (HIL), hole-transport layer (HTL), emission layer, and electron transport layer (ETL), respectively. Before fabricating the devices, the ITO substrates were ultrasonically cleaned with a standard regiment of acetone, ethanol, deionized water, and isopropanol followed by an ex situ UV ozone treatment in air for 5 min. PEDOT:PSS (Baytron PH) was spin-cast from aqueous solution at 3500 rpm for 60 s to form a ~20 nm thickness film. The PEDOT:PSS film was annealed at 110 °C for 30 min in air to obtain a highly conductive layer. The poly-TPD layer was spin-coated onto the PEDOT:PSS layer at 1600 rpm for 1 min from a 0.4 wt.% chlorobenzene solution to give a homogeneous layer of 35 nm. Then, this film was dried at 100 °C in a glove box (MBRAUN) for 30 min. The QD layer was deposited on poly-TPD by spin-coating QD toluene solution (15 mg/ml) at 2000 rpm and then annealed at 70 °C for 30 min in the same glove box (MBRAUN). The ZnO nanoparticles were spin-coated onto the QD layer at 2000 rpm from a 30 mg/ml ZnO butanol solution and then annealed at 70 °C for 30 min in the glove box (MBRAUN) and the thickness is about 40 nm. At last, the AgNW cathode was fabricated by spin-coating the AgNW isopropyl alcohol ink with a concentration of 8 mg/ml. Then, the AgNW cathode with a width of 3 mm was obtained by mechanically scraping the rest AgNWs from the substrate with a sharp scraper. The area of the device pixels were also measured with a microscope to ensure the accuracy of the device efficiency and current density. For the control device, the Al cathode lines with a width of 3 mm were deposited orthogonally to the 3 mm ITO anode lines to form a 9 mm^2^ active area in a vacuum chamber with the pressure below 4 × 10^−6^ Torr.

### Sample Characterization

The characteristics of current–voltage–luminance a were measured by a programmable Keithley model 2400 power supply and a Minolta Luminance Meter LS-110, respectively, in atmosphere conditions without any encapsulation for the devices. The spectra of the devices were obtained through Ocean Optics Maya 2000-PRO spectrometer.

The room temperature absorption/transmittance spectra were measured with an ultraviolet/visible spectrometer (UV 1700, Shimadzu) and the PL spectrum of the QDs in toluene was collected by a Hitachi F-4500 spectrophotometer under an excitation wavelength of 400 nm. The transmission electron microscopy (TEM) images were recorded on a Philips TECNAI G2 and the morphology of ZnO and AgNW films were characterized by scanning electron microscope (SEM) (Hitachi S4800). The sheet resistance (Rs) of AgNW film fabricated on a 2.5 cm × 2.5 cm glass/ZnO nanoparticle substrates was measured through four-point probe.

## Additional Information

**How to cite this article**: Jing, P. *et al.* Vacuum-free transparent quantum dot light-emitting diodes with silver nanowire cathode. *Sci. Rep.* **5**, 12499; doi: 10.1038/srep12499 (2015).
